# lncRNA PCGEM1 Regulates the Progress of Colorectal Cancer through Targeting miR-129-5p/SOX4

**DOI:** 10.1155/2022/2876170

**Published:** 2022-09-20

**Authors:** Bingsheng Guan, Fazhi Chen, Zhenpeng Wu, Cunchuan Wang, Jingge Yang

**Affiliations:** Department of Gastrointestinal Surgery, First Affiliated Hospital of Jinan University, Guangzhou, China

## Abstract

Prostate cancer gene expression marker 1 (PCGEM1) has abnormal expression level in a variety of malignant tumor. However, the relationship between PCGEM1 and colorectal cancer is still unclear yet. This study is aimed at identifying the role of PCGEM1 in colorectal cancer. qRT-PCR was used to examine the expressions of the expression of lncRNA PCGEM1 and SOX4 in CRC tissues and cell lines. The biological functions of lncRNA PCGEM1 and SOX4 were examined by CCK-8 assay, Transwell assay, immunohistochemistry, western blotting, RNA interference, and gene overexpression techniques. Bioinformatics analysis was used to find the potential downstream molecule of PCGEM1 and miR-129-5p. The relationship between PCGEM1, miR-129-5p, and SOX4 was assessed by dual luciferase activity assay. We found that PCGEM1 is overexpressed in colorectal cancer cells and tissues, while miR-129-5p is underexpressed. SOX4 is overexpressed in colorectal cancer cells and tissues. Functionally, PCGEM1 silencing can significantly inhibit the proliferation, invasion, and migration of colorectal cancer cells. Mechanically, PCGEM1 acted as a sponge for miR-129-5p and absorbed its expression, and miR-129-5p was found to target SOX4, constructing the axis of PCGEM1/miR-129-5p/SOX4 in colorectal cancer. In conclusion, PCGEM1 mediates the proliferation, invasion, and migration of colorectal cancer cells by targeting miR-129-5p/SOX4 axis.

## 1. Background

Colorectal cancer (CRC) is a common digestive system tumor nowadays. According to the global cancer statistics, CRC is the third most common cause of cancer occurrence and the second most common cause of cancer-related death in the world [1–3]. Therefore, CRC has made a great negative impact on people's life and property security. Despite many forms of treatment, some patients have a poor prognosis. Understanding the molecular mechanisms of CRC is conducive to better treatment of CRC.

According to the human transcriptome analysis with the Human Genome Project (HGP), only about 2% of human base sequence has the ability to code protein. The remaining 98% of human base sequence can only be transcribed into noncoding RNAs (ncRNAs). A majority of ncRNAs are long noncoding RNA (lncRNA), which has at least 200 base pairs in lengths [4]. Previous research indicated that LncRNAs has a significant relationship with different cancers [5, 6], some work as cancer-promoting lncRNA and some as cancer-suppressing lncRNA [7].

Prostate cancer gene expression marker 1 (lncRNA PCGEM1) was first reported in prostate cancer. However, some researches showed that PCGEM1 has abnormal expression level in a variety of malignant tumors, which can affect the progress of cancer cells, including cervical carcinoma, renal carcinoma, gastric cancer, and endometrial carcinoma [8–11]. The effect of PCGEM1 in CRC has not been well studied yet; we hypothesize that PCGEM1 could participate in regulating the development of colorectal cancer. Therefore, this study is aimed at analyzing the expression of PCGEM1 in colorectal cells and tissues; at analyzing the effect of PCGEM1 on the proliferation, invasion, and migration of colorectal cancer; and at uncovering the mechanisms involved, so as to clarify the role of PCGEM1/miR-129-5p/SOX4 axis in colorectal cancer.

## 2. Methods

### 2.1. Clinical Cases and Tissue Samples

From June 2019 to January 2020, we performed surgical resection for 35 patients diagnosed with CRC in our hospital; these patients did not receive any chemoradiotherapy before surgery. Paired colorectal cancer tissue and adjacent normal tissue were collected from all patients. During surgery, resected tissue samples were immediately frozen in liquid nitrogen and stored at -80°C for further RNA isolation.

### 2.2. Cell Culture

Colorectal cancer cells (HT29, LoVo, SW480) and normal colonic epithelial cell (NCM460) were provided by the Shanghai Institute of Biochemistry and Cell Biology at the Chinese Academy of Sciences. Colorectal cancer cell (HCT116) was provided by the Guangzhou LaiSe Biological Technology Company. Cells were cultured in an incubator (culture conditions include constant temperature of 37°C and 5% CO_2_).

### 2.3. RNA Extraction and qRT-PCR

TRIzol reagent was used to extract the total RNA of colorectal cancer cells and normal cells. The isolated RNA was reverse-transcribed into cDNA and then performed qRT-PCR using the Fluorescence Quantitative Reverse Transcription Kit (Solarbio, Beijing, China). Relative data were normalized to GAPDH. The gene primer sequences were as follows: lncRNA PCGEM1 forward primer, 5′-ACAGCTCCTGGAAGAGGACT-3′, reverse primer, 5′-TTTTCCAAAGGGTCCGCTGT-3′.

### 2.4. CCK-8 Experiment

A Cell Counting Kit-8 was used to examine the proliferation ability of colorectal cancer cells (HCT116, SW480). In each well of the 96-well plate, 100 *μ*l of cell fluid with the logarithmic phase growing cells was added. After culturing in an incubator for 24 h, their absorbance at 450 nm was measured with a microplate analyzer.

### 2.5. Transwell Experiment

Transwell experiment was conducted to examine the invasion and migration ability of colorectal cancer cells (HCT116, SW480). After the fusion of colorectal cancer cells reached 70-90%, the cells were digested and counted by trypsin, and the concentration of the cell suspension was adjusted to 1 × 10^5^/mL in serum-free medium. These cells were transferred to an incubator and cultured for 16-30 hours and then fixed with 4% paraformaldehyde for 15-30 min. The staining solution was gently cleaned with PBS, and the number of cells in different fields was counted under the visual field of the inverted microscope.

### 2.6. Immunohistochemistry

Paired colorectal cancer tissue and adjacent normal tissue were collected. 4% PFA was used for tissue fixation and paraffin-embedded tissue sections. After paraffin removal and flush with PBS fluid, each slice was added the first antibody and the second antibody. And then, ultrapure water was used to rinse the slices, and the color rendering was observed under a color microscope by using DAB color rendering reagent box.

### 2.7. Western Blotting (WB)

After lysed in lysis buffer, cells were transferred to Eppendorf tubes. Bovine serum albumin (BSA) standard dilution buffer was diluted to 500 *μ*g/mL and used to determine the standard curve of protein concentration. The tested sample was added to the 96-well plate and used for colorimetric reaction. Bio-Rad vertical electrophoresis system was used for cataphoresis. After adding the first antibody and the second antibody, chemiluminescence detection kit was used for chemiluminescence protein detection.

### 2.8. Dual Luciferase Activity Assay

LINC00071 (PCGEM1) 3′UTR gene fragment was generated, and then, wild-type PCGEM1 (PCGEM1 WT) and mutant PCGEM1 (PCGEM1 Mut) were cloned into PsichecKTM-2 vector by homologous recombination method. Then, colony PCR and gene sequencing were performed to verify the success of the vector construction. Then, Lipofectamine 2000 was used to introduce the above plasmids together with miR-129-5p inhibitor and its mimics into HCT116 and SW480 cells. After 48 hours, the cells were collected and lysed. The luciferase activity of each group was detected by Promega double luciferase detection kit.

### 2.9. Statistical Analysis

All statistical analyses were performed using the SPSS software 20.0 and graphed using the GraphPad Prism software. Student's *t*-test or one-way ANOVA was used to determine differences between groups. *P* < 0.05 was considered statistically significant.

## 3. Results

### 3.1. Expression of lncRNA PCGEM1 in Colorectal Cancer

**Figure 1:**
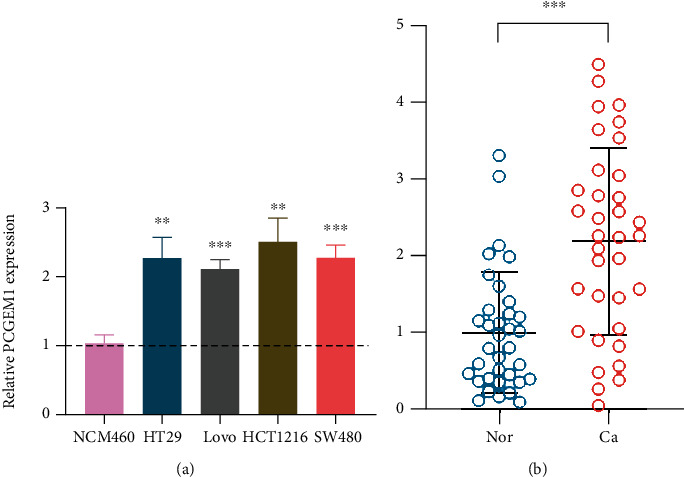
(a) Expression level of PCGEM1 in colorectal cancer cells. (b) Expression level of PCGEM1 in colorectal cancer tissues.

In experiments of cell level, we found that when compared with NCM60, the four colorectal cancer cell lines (HT29, LoVo, HCT116, and SW480) had significant increased expression level of PCGEM1, with the multiples of 2.28, 2.20, 2.51, and 2.28 (Figure 1(a)). Similar results were also found in experiments of tissue level, which indicated that the PCGEM1 expression in colorectal cancer tissues was 2.35 times higher than that in adjacent normal tissue (Figure 1(b)).

### 3.2. Biological Behavior of lncRNA PCGEM1 in Colorectal Cancer

**Figure 2:**
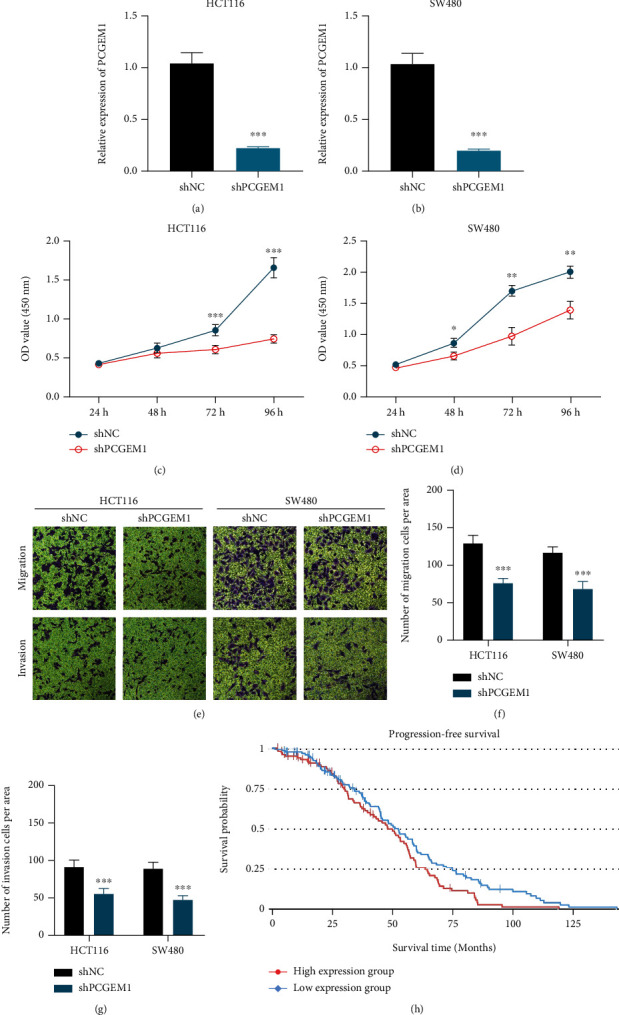
(a–d) Effect of PCGEM1 knockdown on the proliferation of HCT116 and SW480 cells. (e–g) Effect of PCGEM1 knockdown on the invasion and migration of HCT116 and SW480 cells. (h) Survival analysis curve of PCGEM1 and progression-free survival.

In cellular function experiments, cell proliferation curve showed that PCGEM1 knockdown could significantly inhibit the proliferation of HCT116 and SW480 cells (Figures 2(a)–2(d)), which means that PCGEM1 can promote the proliferation of colorectal cancer cells. In addition, the results of Transwell analysis revealed that the invasion and migration ability of colorectal cancer cells were significantly reduced after PCGEM1 knockdown (Figures 2(e)–2(g)).

When considering the relationship between PCGEM1 and patients' survival outcomes, the results from online database lnCAR showed that patients with low PCGEM1 expression had significantly higher progression-free survival than those with high PCGEM1 expression (Figure 2(h)). These results indicate that PCGEM1 serves as an oncogene in colorectal cancer.

### 3.3. lncRNA PCGEM1 Downregulates the Expression of miR-129-5p

Through miRcode software, we found that there were binding sites between PCGEM1 3′UTR and miR-129-5p, which means that miR-129-5p may be the downstream target gene of lncRNA PCGEM1.

**Figure 3:**
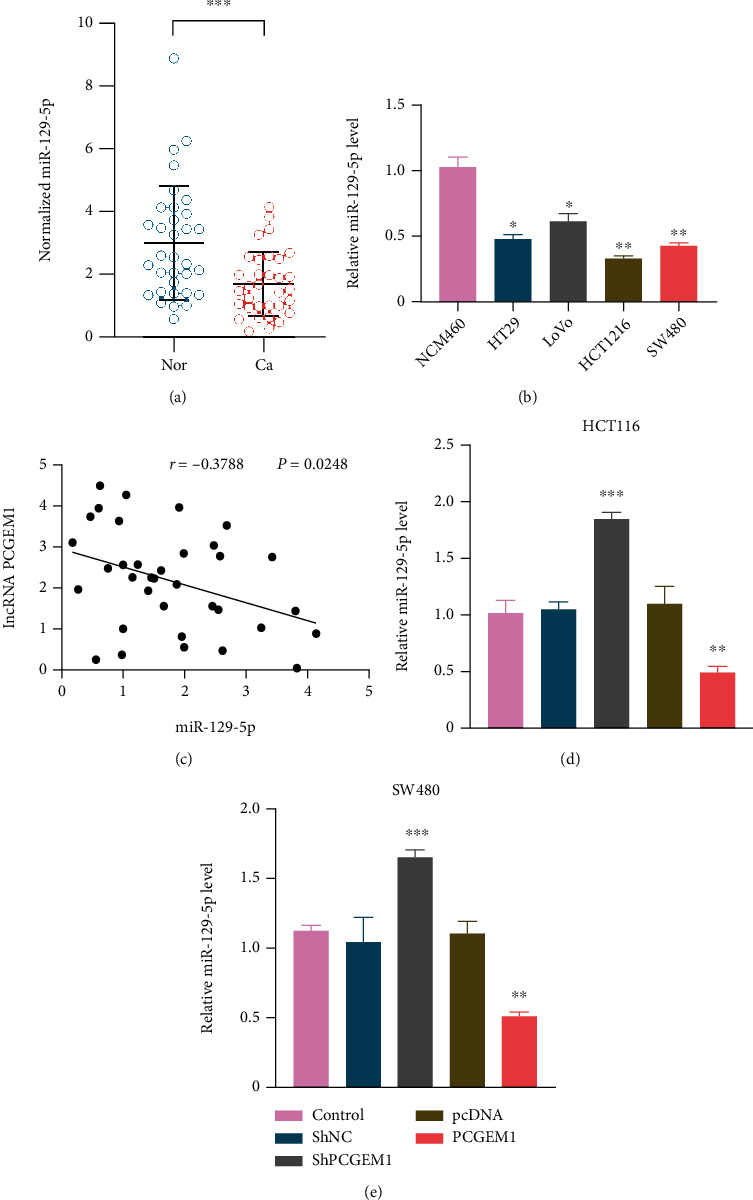
(a, b) Expression level of PCGEM1 in colorectal cancer cells and tissues. (c) Correlation analysis between PCGEM1 and miR-129-5p expression levels. (d, e) Effect of PCGEM1 overexpression and knockdown on miR-129-5p level.

In experiments of cell and tissue levels, it was proved that miR-129-5p is downregulated in colorectal cancer (Figures 3(a) and 3(b)). In addition, the Pearson correlation analysis found a significant correlation between PCGEM1 and miR-129-5p expression levels in colorectal cancer tissue (Pearson *r* = −0.3788, *P* = 0.0248) (Figure 3(c)).

In order to explore whether PCGEM1 can target the expression level of miR-129-5p, RNA interference and gene overexpression techniques were used to detect the effects of PCGEM1 overexpression and knockdown on miR-129-5p (Figures 3(d) and 3(e)); the results indicated that lncRNA PCGEM1 can regulate the expression level of miR-129-5p as a miRNA sponge. Moreover, dual luciferase activity assay was used to verify that miR-129-5p can target binding to PCGEMA.

### 3.4. miR-129-5p Mediates Colorectal Cancer Tumorigenesis by Targeting SOX4

**Figure 4:**
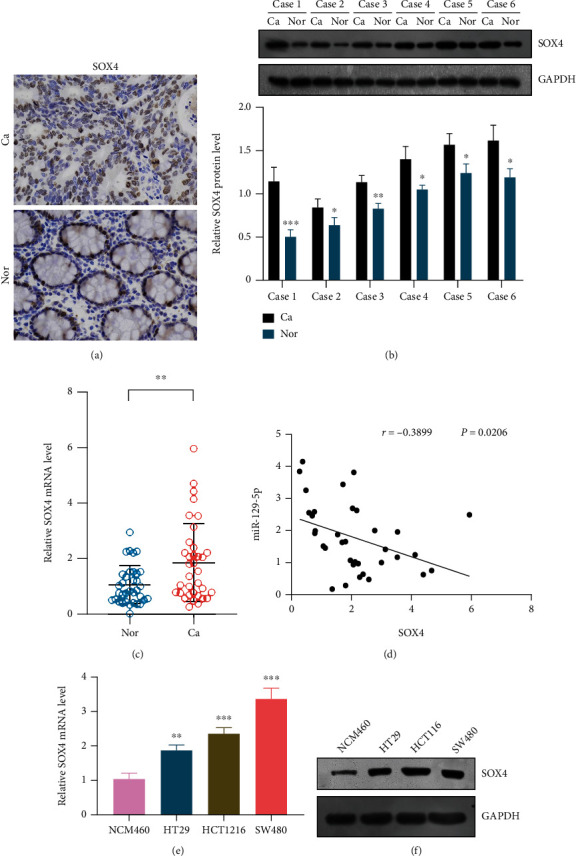
(a–c) SOX4 expression in colorectal cancer tissue detected by immunohistochemistry, western blotting, and RT-qPCR. (e, f) SOX4 expression in colorectal cancer tissue detected by RT-qPCR and western blotting.

The results above showed that PCGEM1 can act as an oncogene in colorectal cancer and targets the expression of miR-129-5p. In order to explore the downstream molecular mechanisms, the TargetScan software was used to find the potential downstream gene, which revealed that SOX4 had some potential binding sites with miR-129-5p. Immunohistochemistry, western blotting, and RT-qPCR found that SOX4 expression was upregulated in clinical colorectal cancer tissue (Figures 4(a)–4(c)). Similar results were also found in colorectal cancer cells (Figures 4(e) and 4(f)).

**Figure 5:**
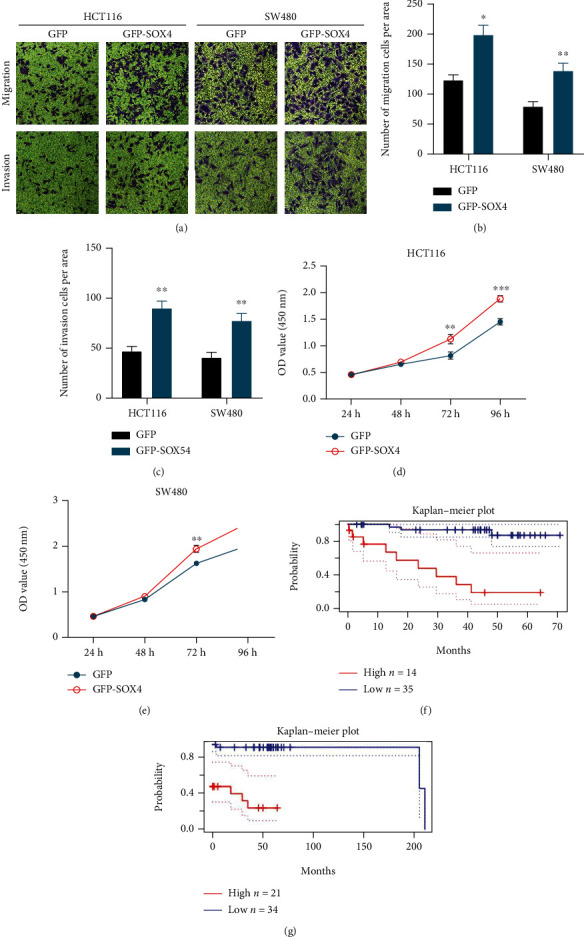
(a–c) Effect of SOX4 overexpression on the invasion and migration of HCT116 and SW480 cells. (d, e) Effect of SOX4 overexpression on the proliferation of HCT116 and SW480 cells. (f, g) Survival analysis curve of SOX4 expression and tumor-free survival and disease-specific survival.

In the study of cellular function, overexpression of SOX4 can significantly promote the proliferation, invasion, and migration of colorectal cell (Figures 5(a)–5(e)). Survival analyses revealed that patients with low SOX4 expression had significantly higher tumor-free survival and disease-specific survival than those with high SOX4 expression (Figures 5(f) and 5(g)).

**Figure 6:**
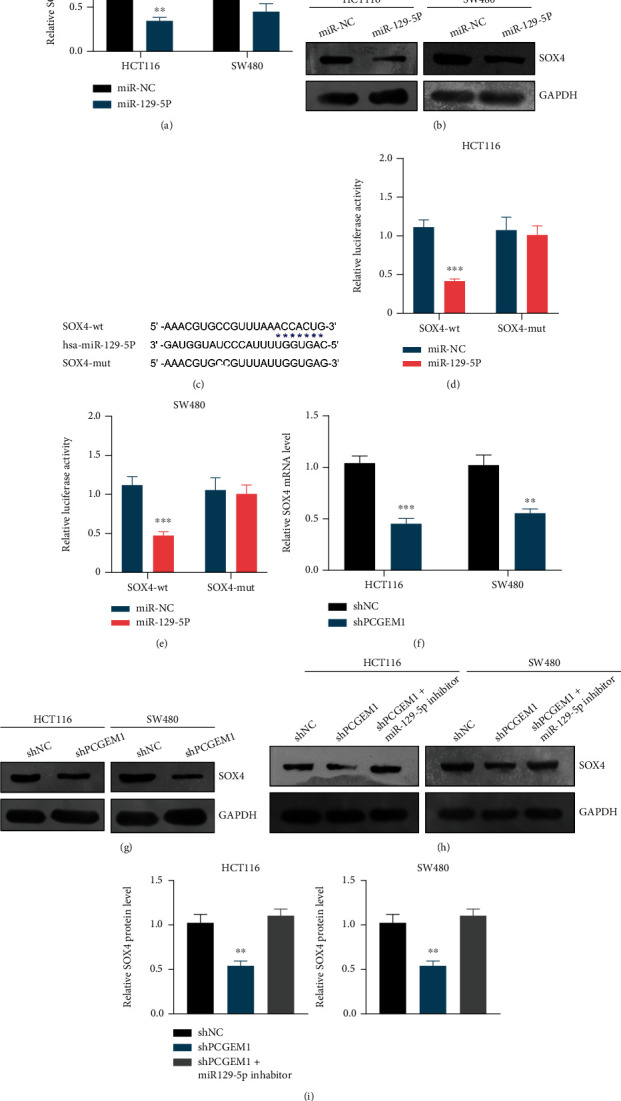
(a, b) Effect of miR-129-5p on SOX4 expression detected by RT-qPCR and western blotting. (c) Dual luciferase activity assay for confirming the relation between SOX4 and miR-129-5p. (d, e) Effect of PCGEM1 knockdown on SOX4 expression detected by RT-qPCR and western blotting. (f) Rescue experiments for confirming the axis of PCGEM1/miR-129-5p/SOX4.

Regarding the relationship between miR-129-5p and SOX4, correlation analysis revealed that miR-129-5p expression was negatively related with SOX4 (Pearson *r* = −0.3899, *P* = 0.0206) (Figure 4(d)). The results of RT-qPCR and western blotting displayed that overexpression of miR-129-5p can significantly inhibit the expression of SOX4 mRNA (Figures 6(a) and 6(b)). Furthermore, dual luciferase activity assay confirmed that SOX4 is a target gene of miR-129-5p (Figure 6(c)).

### 3.5. lncRNA PCGEM1 Upregulates the Expression of SOX4 by Targeting miR-129-5p

In order to explore whether the molecular pathway of PCGEM1/miR-129-5p/SOX4 exists in colorectal cancer or not, we subsequently analyze the effect of PCGEM1 on SOX4, which found that PCGEM1 silencing can significantly reduce endogenous SOX4 mRNA and protein expression in colorectal cancer cells (Figures 6(d) and 6(e)), which would be recovered by miR-129-5p silencing (Figure 6(f)). The results of rescue experiments confirmed that SOX4 served as the functional protein of PCGEM1/miR-129-5p in colorectal cancer.

## 4. Discussion

lncRNA is a hotspot in the study of mechanism of malignant tumor in these years. lncRNA PCGEM1 has been proved to participate in the progress of several cancers. However, the effect and mechanism of PCGEM1 in colorectal cancer is still unknown. A better understanding of the specific therapeutic targets for CRC is essential to advance and improve effective treatment methods.

In this study, dysregulated lncRNA PCGEM1 was found in colorectal cancer tissues/cells and identified to be significantly upregulated. Cellular functional studies showed that lncRNA PCGEM1 silencing inhibited the proliferation, invasion, and migration of colorectal cancer. These results revealed that PCGEM1 can act as a cancer-promoting gene for the progress of PCGEM1. lncRNA and miRNA are both noncoding genes, and lncRNA can act as a miRNA sponge so as to regulate the function of miRNA. For example, previous research results indicated that lncRNA PCGEM1 can regulate the expression of miR-642a-5p in cervical carcinoma [8], the expression of miR-433-3p in renal carcinoma [9], the expression of miR-590-3p in non-small-cell lung cancer [12], and so on.

Bioinformatics analysis is helpful for us to find the potential downstream molecule during the study of mechanism. Using this method, it was found that miR-129-5p is a potential downstream target of lncRNA PCGEM1, which means that lncRNA PCGEM1 can target miR-129-5p as a miRNA sponge. The regulation axis of PCGEM1/miR-129-5p has been reported in gastric cancer and cervical cancer in previous studies [13, 14]. However, our study represents the first publication to confirm this axis in colorectal cancer, with the evidences from correlation analysis and dual luciferase activity assay. In addition, overexpressing and silencing PCGEM1 in colorectal cancer cells could make the expression level of miR-129-5p downregulated and upregulated, respectively, suggesting that PCGEM1 can negatively target miR-129-5p. Regarding the expression level of miR-129 in cancer, the results in literature are inconsistent, with elevated level in oral carcinoma and reduced level in gastric cancer, lung cancer, and breast cancer [15–17]. These differences may be attributed to the fact that different tumors have different internal microenvironments. In our study, decreased miR-129-5p level was found in cellular level and tissue level.

According to the role of miRNA in tumor development, miRNA can be divided into cancer-promoting miRNA and cancer-suppressing miRNA. From the above results, we found that miR-129-5p can act as a cancer-suppressing miRNA in colorectal cancer. Previous research results showed that miR-129-5p has multiple target genes. For example, miR-129-5p can regulate the expression of COL1A1 so as to promote the progression of gastric cancer [17], regulate YWHAB to affect the progression of lung cancer [16], and target ETV1 to inhibit prostate cancer proliferation [18]. But for colorectal cancer, what is the potential target gene for miR-129-5p, bioinformatics analysis was used again in our study, and SOX4 was found.

Upregulated expression of SOX4 in colorectal cancer was identified in our study, which was consistent with previous researches [19]. In addition, overexpressing SOX4 can increase the invasion and migration ability of colorectal cancer cell, which means that SOX4 can act as a cancer-promoting gene for colorectal cancer. In colorectal cancer, SOX4 has been reported as a target gene of some miRNAs, including miRNA-212, miRNA-130a, miRNA-133a, and miRNA-539 [20–23]. However, the axis of miR-129-5p/SOX4 in colorectal cancer has not been reported yet. In our study, cell functional studies and dual luciferase activity assay confirmed the existence of miR-129-5p/SOX4 axis in colorectal cancer. Furthermore, rescue experiments indicated that lncRNA PCGEM1 can regulate colorectal cancer proliferation, invasion, and migration through targeting miR-129-5p/SOX4.

To the best of our knowledge, this study represents the first report to confirm the axis of lncRNA PCGEM1/miR-129-5p/SOX4 in colorectal cancer. However, some limitation should be pointed out. At first, the sample size of clinical patients is not large enough, and more follow-up data need to be collected to further evaluate the relationship between the expression levels of lncRNA PCGEM1, miR-129-5p, and SOX4 in colorectal cancer tissues and patient prognosis. In addition, this study only conducted in vitro cell experiments and clinical tissue detection; in vivo experiments in animal models are needed to further clarify the role of lncRNA PCGEM1/miR-129-5p/SOX4 regulatory axis in colorectal cancer.

## 5. Conclusion

This study finds that lncRNA PCGEM1 and SOX4 are overexpressed in colorectal cancer, while miR-129-5p is underexpressed. PCGEM1 mediates the proliferation, invasion, and migration of colorectal cancer cells by targeting miR-129-5p and regulates the expression of SOX4.

## Data Availability

The analyzed datasets generated during the study are available from the corresponding author on reasonable request.

## Abbreviations

PCGEM1: Prostate cancer gene expression marker 1
CRC: Colorectal cancer
HGP: Human Genome Project
ncRNAs: Noncoding RNAs
lncRNA: Long noncoding RNA
qRT-PCR: Quantitative real-time PCR
CCK-8: Cell Counting Kit-8
WB: Western blotting
BSA: Bovine serum albumin.
